# Assessing the efficacy of amyotrophic lateral sclerosis drugs in slowing disease progression: A literature review

**DOI:** 10.3934/Neuroscience.2024010

**Published:** 2024-04-30

**Authors:** Ubaid Ansari, Meraj Alam, Dawnica Nadora, Zohaer Muttalib, Vincent Chen, Isabel Taguinod, Megan FitzPatrick, Jimmy Wen, Zaid Ansari, Forshing Lui

**Affiliations:** California Northstate University College of Medicine, USA

**Keywords:** amyotrophic lateral sclerosis, neurodegenerative disease, Qalsody, Relyvrio, Radicava, Rilutek, Tiglutik, Exservan, Nuedexta

## Abstract

Amyotrophic lateral sclerosis (ALS) is a fatal and intricate neurodegenerative disease that impacts upper and lower motor neurons within the central nervous system, leading to their progressive destruction. Despite extensive research, the pathogenesis of this multifaceted disease remains elusive. The United States Food and Drug Administration (FDA) has granted approval for seven medications designed to address ALS and mitigate its associated symptoms. These FDA-sanctioned treatments are Qalsody, Relyvrio, Radicava, Rilutek, Tiglutik, Exservan, and Nuedexta. In this review, the effects of these seven drugs on ALS based on their mechanism of action, dosing, and clinical presentations are comprehensively summarized. Each medication offers a distinct approach to manage ALS, aiming to alleviate the burdensome symptoms and slow the disease's progression, thereby improving the quality of life for individuals affected by this neurological condition. However, despite these advancements in pharmaceutical interventions, finding a definitive cure for ALS remains a significant challenge. Continuous investigation into ALS pathophysiology and therapeutic avenues remains imperative, necessitating further research collaborations and innovative approaches to unravel the complex mechanisms underlying this debilitating condition.

## 1. Introduction

Amyotrophic Lateral Sclerosis (ALS), also referred to as Lou Gehrig's disease, is a severe neurodegenerative disease characterized by the gradual deterioration of both upper and lower motor neurons situated in the brain and spinal cord [1]. Regarded as a life-altering diagnosis, ALS lacks any definitive cure. This incapacitating disorder results in weakened muscles, diminished motor abilities, and eventually respiratory insufficiency [1]. Despite extensive research efforts, finding effective treatments to halt or slow the relentless progression of ALS remains an ongoing challenge in the field of neurology [2]. In recent years, several drugs have emerged as potential therapies which aim to delay the advancement of ALS and improve patients' quality of life. Among these pharmaceutical interventions, seven particular medications have gained attention for their distinct mechanisms of action and purported abilities to target various pathways associated with ALS pathology. The Amyotrophic Lateral Sclerosis Functional Rating Scale (ALSFRS) serves as a tool to assess the functional status of individuals diagnosed with Amyotrophic Lateral Sclerosis, allowing for the monitoring of changes in a patient's functionality over the course of time.

The United States Food and Drug Administration (FDA) has currently sanctioned seven medications for addressing ALS and its associated symptoms: Qalsody, Relyvrio, Radicava, Rilutek, Tiglutik, Exservan, and Nuedexta. Qalsody, or tofersen, represents a groundbreaking treatment targeting ALS associated with mutations in the superoxide dismutase 1 (SOD1) gene [3]. Its approval in 2023 marks a pivotal milestone in addressing this specific genetic subtype of ALS. Relyvrio, approved in 2022, is another promising drug that combines sodium phenylbutyrate and taurursodiol to intervene in nerve cell death processes by intercepting stress signals within cells [4]. Radicava, or edaravone, was approved in 2017 [5]. This marked the first novel ALS treatment in over two decades, underscoring its importance in disease management. Rilutek, or riluzole, was the first approved ALS drug in 1995. It remains relevant by inhibiting glutamate release and extending patients' lives by approximately three months [6]. Tiglutik, approved in 2018, introduces a new thickened liquid form of riluzole. It provides an alternative to the traditional oral pill, thereby addressing potential issues related to tablet administration [7]. Exservan, approved in 2019, introduces an oral film formulation of riluzole [8]. It is specifically designed for ALS patients experiencing severe swallowing difficulties. It dissolves upon contact with the tongue to obviate the need for swallowing a pill or liquid [8]. Nuedexta, approved in 2010, is another promising drug that combines dextromethorphan hydrobromide (HBr) and quinidine sulfate to manage pseudobulbar affect (PBA) [9].

In light of the urgent need for effective interventions to alleviate the burden of ALS, a rigorous analysis of these pharmaceutical agents becomes pivotal in guiding research priorities, determining clinical strategies, and improving the lives of individuals affected by this neurological disorder. By exploring these drugs' individual contributions and comparative efficacies, this review aims to contribute to the ongoing discourse regarding the management of ALS.

## 2. Qalsody

Tofersen, sold under the trade name Qalsody, is an intrathecally-administered antisense oligonucleotide that selectively targets mRNA for mutated SOD1 gene products. The SOD1 enzyme catalyzes the conversion of harmful superoxide radicals into oxygen and hydrogen peroxide to reduce cellular damage, and potentially plays a role in nuclear gene transcription activation in response to oxidative stress [10]. SOD1 mutations are the second leading cause of familial ALS, accounting for approximately 10–20% of cases, and can be seen in 1–2% of sporadic ALS cases. Toxic gain of function and possible loss of function mutations may be implicated in the pathogenesis of ALS due to SOD1 (SOD1-ALS), as astrocytes and microglia expressing mutant SOD1 proteins demonstrate toxicity to motor neurons which contributes to the neuronal degeneration seen in this disease [11]. Qalsody is designed to mediate the degradation of SOD1 mRNA by forming an RNA-DNA hybrid with the mRNA that is then hydrolyzed by the ubiquitously expressed enzyme ribonuclease H, ultimately downregulating synthesis of the mutated SOD1 gene products [12],[13].

Treatment with Qalsody typically begins with three loading doses once every fourteen days, administered as a 100 mg per 15 mL injection performed by a healthcare professional; afterwards, it is administered once every 28 days [14]. A phase I/II trial evaluating the safety effects of Qalsody in a cohort of 50 people with SOD1-ALS determined that Qalsody decreased SOD1 protein concentrations in cerebrospinal fluid (CSF) by up to 36% in the high-dose group and 3% in the low-dose group compared to placebo in a 12 weeks period. The most common adverse effects were related to the drug's lumbar puncture administration and displayed mild to moderate severity, including headache, dizziness, and post-lumbar puncture syndrome [15]. A subsequent phase III efficacy extension of the same trial conducted with 108 people over 28 weeks determined that though CSF SOD1 protein concentrations decreased by up to 29%, there was no statistically significant decrease in disease progression, which was measured utilizing the ALSFRS-R score in both the placebo and Qalsody study groups [13].

A separate phase III study into the efficacy of Qalsody treatment for presymptomatic SOD1 mutation carriers was initiated in 2021. In this study, a total of approximately 150 participants with elevated plasma neurofilament light chain levels, indicating upper motor neuron damage in ALS, will receive Qalsody dosages for up to two years. Referred to as the ATLAS study, its primary objective is to determine the percentage of participants who develop ALS symptoms within a year of initiating treatment, with secondary objectives involving the evaluation of ALSFRS-R, the time for symptom onset, and the monitoring of adverse reactions. The study will be completed by August 2027 and will determine the efficacy of Qalsody in delaying ALS onset and/or disease progression [16].

## 3. Relyvrio

Relyvrio is an oral-fixed dose combination of taurursodiol and sodium phenylbutyrate, a bile acid and pan-histone deacetylase (HDAC) inhibitor, respectively. Currently, the drug's exact mechanism of action is not well understood. Though it is unknown how the drug exerts its therapeutic effect, it has been established that these two compounds work in conjunction to prevent nerve cell death by inhibiting stress signals within the cell's mitochondria and endoplasmic reticulum [17]. Taurursodiol alone is commonly used to treat chronic cholestatic liver diseases and gallstones due to its anti-apoptotic effects, which includes regulating the expression of specific targets of apoptosis and mitochondrial membrane stabilization [18]. It has been previously demonstrated that taurursodiol also improves energy generated in the mitochondria [19]. Sodium phenylbutyrate operates through another mechanism, increasing the activation of chaperone proteins and thereby decreasing stress in the endoplasmic reticulum [20]. In animal studies, administration of sodium phenylbutyrate has been shown to improve cell survival. In addition, as a HDAC inhibitor, phenylbutyrate is able to regulate transcription, preventing proteins from forming clumps that may lead to nerve cell death and allowing cells to acquire their normal shape [18]. Common notable adverse effects of this drug include diarrhea, abdominal pain, nausea, and upper respiratory tract infections. Taurursodiol may worsen diarrhea in patients with comorbidities that interfere with bile acid metabolism.

Relyvrio is the most recent medication approved for ALS treatment. It is formulated as a room-temperature oral suspension. The recommended dosage is 3 grams of sodium phenylbutyrate and 1 gram of taurursodiol taken once a day for the first three weeks, then twice a day thereafter [21].

Administration of sodium phenylbutyrate leads to improved cell survival as observed in animal studies. Since sodium phenylbutyrate inhibits HDAC, it is able to regulate transcription resulting in normal protein aggregation. Moreover, an increase in blood histone acetylation levels have been demonstrated in a 2020 clinical trial for this drug [22]. A phase II multicenter, randomized, double-blinded trial tested the safety and efficacy of a fixed-dose combination in 137 participants that were previously taking riluzole. The participants were placed in either the placebo group or the Relyvrio treatment group for 24 weeks. The study showed that the participants in the treatment group experienced a slower disease progression as measured by the ALSFRS-R by about 25% compared to those in the placebo group [22]. In addition, supplementation of Relyvrio resulted in prolonged median survival of about 6.5 months when compared to the placebo group. Though this trial had a limited number of participants, the drug was approved for ALS due to the disease's severity and a scarcity of safe and effective treatments [22]. Thus, future studies are warranted to further investigate the efficacy and long-term effects of this current drug.

## 4. Radicava

Though no one etiology of ALS has been definitively proven, the evidence for the involvement of oxidative stress is strong [23],[24]. Elevated levels of 3-nitrotyrosine (3-NT), a specific marker of nitro-oxidative neuronal degeneration, have been observed in the lumbar and thoracic spinal cords of ALS patients [23]–[25]. In 2017, edaravone, sold under the trade name Radicava, was approved by the FDA as the first treatment to target this specific etiology [23].

While its exact mechanism is unclear, edaravone is known to be a potent free radical scavenger, particularly of hydroxyl radicals [23]. In one study, edaravone effectively prevented lipid peroxidation by scavenging hydroxyl radicals [26]. Another study showed that edaravone was able to reverse the cytotoxic effects of H2O2 on the viability of cells [27]. SH-SY5Y dopaminergic cells treated with H2O2 had significantly elevated rates of apoptosis. This effect was largely reversed when H2O2 was co-administered with edaravone. Interestingly, proteomics analysis in the same study demonstrated that edaravone also greatly upregulated peroxiredoxin-2 (PRX-2), part of a family of antioxidant enzymes [27],[28]. PRX-2 is known to protect neuronal cells by inhibiting the apoptosis signal regulating kinase signaling cascade [27].

In 2001, a phase II clinical trial was conducted with 20 subjects, with results suggesting that edaravone was safe and effective for the treatment of ALS [29]. In the open-label study, subjects were administered 60 mg of edaravone intravenously once per day for 2 weeks, followed by a 2-week observation period. This 4-week cycle was repeated six times. The decrease in the ALSFRS-R score was significantly less during the 6-month treatment period than during the preceding 6 months.

A phase III double-blind study was initiated in 2006 to confirm the findings in the phase II trial [30]. 206 subjects were recruited and intravenously administered either placebo (n = 104) or edaravone (n = 102) once per day for the first 14 days in cycle 1, and for 10 of the first 14 days during cycles 2 to 6. The researchers found that the reduction in ALSFRS-R score was smaller in the treatment group but did not demonstrate efficacy of treatment. A post-hoc analysis of the study revealed that edaravone had a greater effect among patients who met the following criteria: definite or probable diagnosis of ALS, disease duration less than 2 years, at least 2 points on all items of ALSFRS-R, and forced vital capacity of at least 80% [31].

As post-hoc analyses have limitations on interpretability, another phase III study was initiated in 2011 to substantiate the findings [32]. This was conducted with a similar trial design, but screened for subjects meeting the above criteria. In total, 134 patients were enrolled in the study. The trial found that edaravone was efficacious in a small subset of patients who met the criteria described in the post-hoc analysis, with the treatment group showing a significantly smaller decrease in ALSFRS-R score compared to the placebo group. The researchers also found no difference in the level and frequency of adverse events between the two groups. However, there was no indication that edaravone might be effective in the wider population of ALS patients who do not meet the criteria.

As of 2021, there are four active clinical trials focused on establishing edaravone's safety and identifying associated biomarkers. One clinical trial is also exploring the safety and efficacy of orally administered edaravone [23].

## 5. Rilutek

Riluzole, sold under the trade name Rilutek, is a synthetic benzothiazole that has been used clinically in the management of ALS since its approval by the FDA in 1995 [33]. It was the first of seven drugs to receive FDA approval, and a generic version of riluzole was brought to market in 2013. Riluzole is widely available and is taken as an oral 50-mg tablet twice daily. While it is generally well-tolerated, it should be used with caution in the elderly and in those with hepatic dysfunction because of its association with elevated liver function tests. Although there is no cure for ALS, riluzole is believed to extend survival time and time to tracheostomy in this patient population.

Riluzole's exact mechanism of action is not fully understood due to its array of molecular targets, but its neuroprotective effects in ALS are thought to be associated with the drug's ability to antagonize glutamate transmission in the central nervous system. Riluzole's inhibitory effect on glutamate signaling is important because much of the motor neuron loss characteristic of ALS occurs due to excessive glutamate exposure. The hypothesis of glutamate excitotoxicity proposes that glutamate release from presynaptic nerve terminals will overstimulate N-methyl-D-aspartate (NMDA) receptors that bind glutamate on the postsynaptic neuron [34]. Nerve cells then respond to this stress by inducing free radical production, promoting protein aggregation, and upregulating other mechanisms that eventually result in the neurodegeneration indicative of ALS.

Riluzole employs several processes to counteract the excitotoxicity that leads to neuronal cell death. For one, riluzole will reversibly inhibit voltage-gated sodium channels in presynaptic neurons of the central nervous system, mitigating the inward Na^+^ current needed to drive cell depolarization and eventual vesicular release of the glutamate neurotransmitter via exocytosis [35]. In blocking these sodium channels, riluzole indirectly reduces the hyperexcitability implicated in ALS pathogenesis because it limits the amount of extracellular glutamate that can be present to act on NMDA receptors. While riluzole does not directly affect voltage-gated inward calcium channels in cortical neurons, it has been shown to modulate the late potassium current through a similar reversible inhibition of voltage-activated channels [35].

Riluzole also modulates glutamate signaling during cellular stress by increasing heat shock protein (HSP) expression (namely HSP70 and HSP 90) and upregulating the primary astrocytic glutamate transporter: Excitatory amino acid transporter 2 (EAAT-2) (GLT-1 in rodent models) [36]. EAAT-2 is abundant in the brain and plays a key role in removing glutamate from the synapse when functioning normally. Decreased expression levels of this glial transporter can lead to buildup of extracellular glutamate and the excitotoxicity-mediated neurodegeneration eventually seen in ALS [37]. Treatment with riluzole, however, allows for continued glutamate uptake to prevent this neurotoxicity from ever occurring [36].

Clinically, riluzole has been shown to be efficacious in slowing symptom progression, lengthening time to tracheostomy, and extending overall survival time by approximately 3 months [38],[39]. A placebo-controlled, double-blinded clinical trial aimed at evaluating riluzole's effectiveness at improving survival and delaying functional impairment in ALS patients took place in France in 1990 [38]. A cohort of 155 ALS patients were enrolled in the study, with 78 patients assigned to the placebo group and 77 patients given 100 mg of riluzole each day. After twelve months of treatment, 74% of patients in the treatment group were alive, compared with 58% of patients in the placebo group. In addition to the statistically significant survival advantage seen with riluzole treatment (p = 0.014), the researchers demonstrated how riluzole may confer an advantage in prolonging functional status in ALS patients, as the rate of muscle strength deterioration was significantly slower in the treatment group compared to the placebo group (p = 0.028).

Riluzole is the only drug therapy available for ALS patients in Europe [40]. However, this treatment protocol may change as a decision regarding market authorization of the tyrosine kinase inhibitor Masitinib is expected to be made in early 2024 [41]. Masitinib showed promising results as an adjuvant to riluzole in a phase II/III, randomized double-blind clinical trial of 394 patients that took place from 2013–2015 [42]. Results from this study demonstrated how masitinib led to a slower rate of functional decline for ALS patients when given orally with riluzole in 4.5 mg/kg daily doses compared to when patients were only given riluzole. This suggests the potential use of masitinib as an adjunct therapy to delay the progressive physical and cognitive impairments in ALS patients. Continued exploration of drug therapies that can be co-administered with riluzole to improve patients' functional status and prolong life expectancy is essential to improve overall outcomes for the ALS community.

## 6. Tiglutik

Tiglutik is a homogenous oral suspension variant of riluzole that is provided as a twice daily 5 mg/ml dosage every 12 hours that provides the usual dose of 100 mg of oral riluzole. The texture of Tiglutik is classified as a level two (nectar-like) that is considered mildly thick according to the International Dysphagia Diet Standardization Initiative (IDDSI). Thick fluids allow a safer intake for patients with dysphagia. It also utilizes a novel flocculation technology that decreases the local anesthetic effects of oral intake in the mouth. This formulation provides an additional benefit of increased compliance and accuracy of dosing due to the ease of administration. Additionally, Tiglutik is composed of saccharin, antifoam, and aluminum magnesium silicate which provides the physical and chemical stabilization of this liquid formulation [43],[44]. It is also bioequivalent when administered orally or intragastrically thus it is safe to be administered both orally and through percutaneous endoscopic gastrostomy (PEG) feeding tubes which overcomes the limitations of traditional oral tablet formulations [45]. The maximum plasma concentration of oral suspension is also ~20% greater than the tablet formulation.

The rationale for developing a liquid formulation is evident as the other alternative previously was to crush riluzole tablets for patients with dysphagia. This option comes with severe possible complications such as dosage errors due to decreased absorption, risk of aspiration pneumonia, and anesthetic effects on the tongue [46].

Dysphagia is a very common ALS complication and can be seen in over 80% of patients in the later stages of the disease. It is associated with worsened clinical outcomes, malnutrition, weight loss, aspiration pneumonia, and decreased adherence to medications [44]. Additionally, patients may present with silent dysphagia, thus early detection and management is essential [47]. The liquid formulation provides a benefit for patients that have dysphagia or have undergone PEG to continue riluzole treatment. It is approved for usage in all stages of ALS and is associated with improved compliance, reduced need to switch medication as dysphagia progresses, prolonging the need for PEG, and ease of administration. Tiglutik should be considered over the oral tablets both as first-line treatment for patients at diagnosis as well as a secondary treatment for patients with dysphagia. However, future studies are required to better compare the efficacy and long-term effects compared to the tablet form.

## 7. Exservan

Exservan, also known as oral film riluzole, is an oral film form of the drug that can be administered without the use of water, and is valuable for patients who are having symptoms of dysphagia or difficulty swallowing [48]. The exact mechanism of action for Exservan remains unclear;, yet, it is believed to be associated with glutamate signaling [8]. Glutamate is an important excitatory neurotransmitter that helps with the transmission of nerve signals [36]. However, excess glutamate can lead to death of nerve cells [36]. Exservan has been observed to diminish glutamate levels, thereby decreasing the likelihood of nerve cell mortality [8]. Additionally, it was seen that Exservan reduced the accumulation of toxic TAR DNA-binding protein (TDP-43) proteins in nerve cells, a hallmark of the ALS disease which is seen in 97% of patients [49].

Exservan proves particularly beneficial during advanced stages of ALS disease progression, where dysphagia symptoms become more frequent and severe. The oral film is administered onto the tongue's surface, where it dissolves and is ingested as the patient swallows naturally. Exservan is usually taken twice daily before meals at 50 mg dosages. Adverse side effects associated with Exservan comprise oral hypoesthesia, asthenia, nausea, reduced lung function, hypertension, and abdominal pain [50]. Exservan's approval in the United States was supported by data from two clinical trials of riluzole by the FDA. In these trials, a tablet formulation of riluzole significantly extended the time to death or to require a tracheostomy. Researchers looking at the bioequivalence of riluzole oral film and riluzole also found no significant difference between the two drug options under fasting conditions in tested patients [8]. Another study found that there was no deterioration of swallowing function after the administration of riluzole oral film in ALS patients [8]. These trials helped to show how valuable Exservan can be as an oral film to help patients struggling with taking oral tablets on a consistent basis and the effectiveness of Exservan as an alternate drug choice for patients with ALS [8]. Exservan is a valuable formulation of riluzole that is of great significance for patients suffering from amyotrophic lateral sclerosis, especially those in the later stages of the disease. Further research is needed to compare the effectiveness between the oral film solution and the tablet form of riluzole among affected patient groups.

## 8. Nuedexta

Nuedexta is an orally administered combination of Dextromethorphan (20mg) and Quinidine Sulfate (10 mg). Dextromethorphan is an NMDA receptor antagonist that is commonly used as a cough suppressant along with being able to treat PBA [51]. PBA manifests as recurrent, involuntary, and sometimes abrupt bouts of crying or laughing, often disproportionate to the individual's emotional state [9]. Quinidine is a class 1a antiarrhythmic drug that is commonly used to block cardiac sodium channels to prolong action potential duration through QT prolongation and QRS segments [52]. Quinidine inhibits cytochrome P450 2D6 (CYP2D6) which plays a role in the oxidative metabolism of dextromethorphan. Inhibiting CYP2D6 increases the bioavailability of dextromethorphan which can exert its anti-PBA effects [53]. The PBA effect is a disinhibition syndrome in which pathways involving serotonin and glutamate are disrupted. Glutamate is an excitatory neurotransmitter that can explain the involuntary actions that occur with PBA. The cerebellum also seems to play a bigger role in PBA via pathways from the cortex to the pons to the cerebellum that control cognition and affective function alongside motor function [54]. The exact mechanism by which Nuedexta decreases PBA effects is not known. However, one of the possible mechanisms may be due to the binding of sigma-1 receptors in the brain which may be involved in the behavioral aspects commonly seen with PBA [53].

Randomized double-blind clinical trials have been conducted to test the efficacy of Nuedexta in PBA effects. One such study was a 29-day randomized, double-blind Phase III study with 140 ALS patients which found that the drug regimen helped improve PBA symptoms as indicated by Center for Neurologic Study Lability Scale (CNS-LS) scores [53]. The CNS-LS score is determined by a 7-item questionnaire with a score of 13 or higher suggesting PBA. The researchers found that Nuedexta significantly improved the CNS-LS scores by at least 13 points compared with using dextromethorphan or quinidine alone. Patients at risk of rhythmic heart problems such as QT interval prolongation or AV block should avoid the usage of Nuedexta due to Quinidine's risk profile of QT prolongation which can precipitate torsades de pointes [53]. Moreover, patients who are taking drugs that can affect serotonin levels such as selective serotonin reuptake inhibitors (SSRIs) and monoamine oxidase inhibitors (MAOI) should avoid Nuedexta due to dextromethorphan's effects on serotonin levels, which can potentially lead to serotonin syndrome [53].

## 9. Conclusion

The landscape of Amyotrophic Lateral Sclerosis management has witnessed significant advancements with the approval of seven different medications by the United States FDA. Each of these drugs represents a unique approach in targeting various aspects of ALS pathology. In the ever-evolving landscape of ALS research, the FDA's approval of these various medications reflects ongoing progress in the pursuit of effective ALS treatments. These drugs, each with different mechanisms of action, signify a collective effort to alleviate the burden of this neurodegenerative disease. From inhibiting glutamate release to addressing specific genetic subtypes, these interventions offer a spectrum of approaches to managing ALS symptoms and potentially slowing disease progression. As the complexities of ALS are navigated, it becomes evident that the significance of these approved medications lies not only in their individual contributions but also in the larger context of advancing our understanding of the disease. Additionally, the approval of new treatments and the refinement of existing ones underscore the commitment to enhancing the quality of life for individuals affected by ALS, and highlights the strides made in ALS research that offer not only symptomatic relief but also potential avenues for slowing the disease's progression. However, it is crucial to acknowledge that the urgent need for effective interventions persists, and ongoing research and clinical trials will be crucial in refining existing treatments and discovering new therapeutic approaches. Against the backdrop of ALS inquiry, ongoing explorations delve into emerging modalities and encompass diverse approaches, hoping to present potential transformative implications in ALS therapy. This review aimed to contribute to the discussion surrounding ALS management by providing insights that can guide research priorities and clinical decision making. These strategies will improve the lives of those affected by this debilitating neurological disorder.
